# Neuroprotection of Gueichih-Fuling-Wan on cerebral ischemia/ reperfusion injury in streptozotocin-induced hyperglycemic rats via the inhibition of the cellular apoptosis pathway and neuroinflammation

**DOI:** 10.7603/s40681-016-0021-5

**Published:** 2016-11-14

**Authors:** Yuh-Fung Chen, Kuo-Jen Wu, Wei-Shih Huang, Yow-Wen Hsieh, Yu-Wen Wang, Huei-Yann Tsai, Ming-Ming Lee

**Affiliations:** 1 Department of Pharmacology, China Medical University, 404 Taichung, Taiwan; 2 Department of Pharmacy, China Medical University Hospital, 404 Taichung, Taiwan; 3 Department of Neurology, China Medical University, 404 Taichung, Taiwan; 4 Department of Neurology, China Medical University Hospital, 404 Taichung, Taiwan; 5 Department of Pharmacy, China Medical University, 404 Taichung, Taiwan; 6 Department of Health and Nutrition Biotechnology, Asia University, Taichung, 413 Taichung, Taiwan; 7 Department of Pharmacology, College of Medicine, China Medical University, No. 91 Hsueh-Shih Road, 404 Taichung, Taiwan

**Keywords:** Gueichih-Fuling-Wan, Cerebral Ischemia/ Reperfusion Injury, Hyperglycemia, Cellular Apoptosis, Neuroinflammation

## Abstract

Background: Risks of stroke link with complications of hyperglycemia. Gueichih-Fuling-Wan (GFW), according to Chinese Medical Code literature, has the promotion of blood circulation and attenuates the swollen plot. Recent pharmacological studies have pointed out its efficacy in patients with cerebral ischemia or diabetes. Therefore, this study determined whether GFW has the protection against cerebral ischemia/ reperfusion (I/R) injury in streptozotocin (STZ)-induced hyperglycemic rats and LPS-induced inflammation in BV-2 microglial cells.

Methods: Extracts of GFW were filtered and frozen to dry for use. Hyperglycemia was induced by intraperitoneal injection of 70 mg/kg STZ. Fourteen days after STZ injection, GFW (1, 2 and 4 g/kg) was orally administered once daily for seven days. Rats were subjected to cerebral ischemia/reperfusion and sacrificed for infarction analysis and neuronal apoptosis detection twenty-one days after STZ injection. MTT assay was used for cell viability; nitrite quantification and western blot analysis of iNOS and COX-2 were used to explore the effects of GFW on LPS-induced inflammation in BV-2 microglial cells.

Results: GFW significantly ameliorated cerebral infarction while dosage was more than 1 g/kg (by 38.03% at 2 g/kg and 52.44% at 4 g/kg), and attenuated neurological deficits by 23.48% (at 2 g/kg) and 47.25% (at 4 g/kg). Furthermore, GFW (2, 4 g/kg) notably decreased TUNEL- and cleaved caspase-3-positive cells in the immunohistochemical stain (*P* < 0.01 and *P* < 0.001, respectively). GFW remarkably increased in Bcl-2 and decreased in caspase-3 and Bax/Bcl-2 ratio protein expressions by Western blot. GFW (0.25, 0.5, 1 mg/ *ml*) significantly reduced LPS-induced NO production in BV-2 microglial cells. And GFW attenuated iNOS and COX-2 expression in LPS-treated BV-2 cells. Conclusions: In summary, GFW has good bioactivities to protect cerebral I/R injury in hyperglycemic rats, which might be due to inhibition of cellular apoptosis and neuroinflammation.

## 1. Introduction

Atherosclerosis in the carotid and vertebrobasilar artery causing cerebral infarction is the chief cause of stroke worldwide [1].

Stroke, the leading cause of and disability and death, is a multifactor disease that forms a possible end state for diabetes, atherosclerosis, and hypertension [2]. Statistically, ischemic stroke is significant major stroke damage, which induces the reduction of oxygen and glucose leading to neuronal excitotoxicity [3, 4]. The previous study reported that diabetic rats might be more susceptible to the cerebral vascular accident [5]. Increased likelihood of mortality and reduced functional recovery in acute hyperglycemia was higher than non-diabetic patients after ischemic stroke [6]. Epidemiological studies found diabetics 2-3 times as often as non-diabetics, a stroke rate twice that of the general population [7-9]. Patients with diabetes in the environment of high blood sugar are likely to suffer atherosclerosis and reduced blood flow, which lead to ischemia and cell death in the ischemic area. Diabetes alters central nervous system dysfunction associated with cognitive change and function abnormality, which increases the performance of reactive oxygen species and inflammatory mediators, and spawns neuronal apoptosis and neurodegeneration after reperfusion injury. It would exacerbate the likelihood of stroke damage [10-12]. Treatment options for stroke nowadays are limited and affect mainly only symptoms. Therapeutic applications are relevant for preventing or inhibiting neurological cell death for a variety of neurodegenerative conditions including ischemia, and stroke.

Inflammation contributes to stroke-related brain injury in both the core and the ischemic penumbra. It is believed to be especially dangerous after reperfusion. Numerous evidences show that post-ischemic inflammation induces the deleterious damage of neuronal cells after stroke, and the activation of microglia, in particular, has been thought as the main contributor by releasing proinflammatory and neurotoxic factors. LPS (lipopolysaccharide), the polysaccharide component of the cell wall of gramnegative bacteria, is the most frequently used model to investigate the inflammatory responses of microglia [13-15]. Stimulation of microglia with LPS induces the release of nitric oxide (NO)-mediated neuron death *in vitro* [16]. Inducible NO synthase (iNOS) is quickly transcribed and expressed in microglia after stimulation with bacterial LPS and cytokines [17]. Cyclooxygenase-2 (COX-2) also plays a predominant role in inflammation [18]. Excess oxygen free radicals could directly generate and damage cerebral tissue through the activation of apoptotic and necrotic cell signaling pathways. The molecular mechanisms of inflammation induced by LPS in microglial cells have been well reported [19- 23]. In most neurodegenerative diseases, the neuronal loss is due to apoptosis. It could particularly be a possible mechanism for hyperglycemia-induced neuronal cell death, which is a successive occurrence of processing including condensation of chromatin, shrinkage of cell and nucleus, membrane bleb, and DNA fragmentation. Therefore, to interrupt the signaling networks that link to neuronal damage to apoptotic degradation is a possible treatment option in neurodegenerative disease [24-26]. Caspase enzymes and Bcl-2 family, two major families of proteins, are the key elements in apoptosis. Caspase-3 plays a pivotal role in apoptosis [27], whereas, there are two classes in Bcl-2 family to regulate apoptosis: the antiapoptotic proteins, including Bcl-2, Bcl-xL and proapoptotic proteins, including Bax and Bak [28].

Gueichih-Fuling-Wan (abbreviated as GFW), a traditional Chinese herbal medicine, has mainly treated gynecological diseases for thousands of years. GFW has the neuroprotective effect in diabetic rats through reducing advanced glycation end products (AGEs) accumulation and oxidative stress [29]. GFW also could improve vascular function and hemorrhological factors in spontaneously diabetic rats [30]. Recent studies show that GFW had the neuroprotective effect against cerebral ischemia in rats [31], and ameliorated memory deficits and neuronal apoptosis in streptozotocin-induced hyperglycemic rodents [32]. No paper has been reported the neuroprotective effect of GFW on cerebral ischemia injury in hyperglycemic rats. Therefore, this study explored the neuroprotective mechanism of GFW in STZ-induced hyperglycemic rats with an ischemia/reperfusion brain injury *in vivo* and LPS-induced inflammation in BV-2 microglial cells *in vitro*.

## 2. Materials and methods

### 2.1. Reagents

Streptozotocin (STZ) was purchased from Sigma-Aldrich (St. Louis, MO), Zoletil^®^ from Virbac Laboratories (Carros, France). BCA protein assay kit was from Thermo Fisher Scientific (Lafayette. CO). NovoLink Polymer Detection System Kit was from Leica Microsystems Inc. (Newcastle Upon Tyne, UK). Apo- BrdU-IHCTM *In situ* DNA Fragmentation Assay Kit was from BioVision (Milpitas, CA); anti-Caspase-3, anti-Bcl-2, anti-Bax and anti-β-actin antibodies from Santa Cruz Biotechnology (Santa Cruz, CA).

### 2.2. Extraction of GFW

Gueichih-Fuling-Wan (abbreviated as GFW), a traditional Chinese remedy, consists of five medicinal herbs in equal proportions: Cinnamomum cassia Blume; Paeonia lactiflora Pall; Paeonia suffruticosa Andr, Poria cocos (Schw.) Wolf; and Prunus persica (L) Batsch. Each medicinal herb (200 g) was extracted twice with 2 L boiling distilled water for two hours. Extracts were filtered and frozen to dry. The yield of extracts was 11.54 %, and GFW freshly prepared in distilled water before experiments.

### 2.3. Ethics statement

The experimental protocol was approved by the Institutional Animal Care and Use Committee of China Medical University (permit number 99-12).

### 2.4. Animals and drug administration

**Figure 1:**
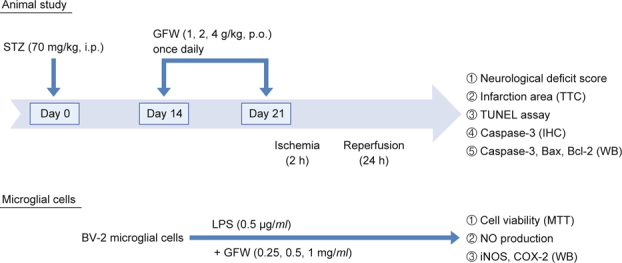

Male Sprague-Dawley (SD) rats with body weight 225-275 g were purchased from BioLASCO Co., Ltd. (Taipei, Taiwan). Animals, fed with regular chow, were housed in standard cages. The room temperature is at constant 22 ± 1°C; relative humidity is 55 ± 5% and with 12 h inverted light-dark cycle. A minimum number of animals and duration of observations required for consistent data were used, rats randomly allocated into four groups (n = 6/group): hyperglycemia, hyperglycemia treated with GFW (1 g/kg, 2 g/kg, 4 g/kg, p.o.). Hyperglycemia was induced by intraperitoneal injection of streptozotocin (STZ) (Sigma; St. Louis, MO) at 70 mg/kg [33]. Three days after STZ injection, overnight fasting and plasma glucose was sampled from animal tail venous blood, and determined by using an automatic glucometer (ACCUCHEK Active, Roche Diagnostics Ltd.; Mannheim, Germany). Those animals, with plasma glucose level higher than 300 mg/dl, were considered for diabetes [32]. The day of STZ injection was designated Day 0. Fourteen days after STZ injection, GFW was orally administered once daily for seven days. Rats were subjected to cerebral ischemia/reperfusion and sacrificed for infarction analysis and neuronal apoptosis detection twenty-one days after STZ injection. GFW (1, 2 and 4 g/kg) was given orally, as shown in Fig. 1.

### 2.5. Surgical procedures of cerebral ischemia/reperfusion

Male SD rats were deeply anesthetized by an injection of 50 mg/ kg of zoletil^®^ (i.p.). Surgical cerebral infarction and ischemia/ reperfusion were as previously described [34]. The rat was placed supine and both common carotid arteries were exposed and were tied off with a plastic line (0.1 mm in diameter). The different concentrations of distilled water, GFW (1, 2, 4 g/kg) are orally administered once daily for seven days before 3-vessels occlusion in rats, respectively. After 90 min of occlusion following by reperfusion for 24 h, the brain of each rat was removed after transcardial perfusion of 0.9% NaCl and 4% paraformaldehyde. Each brain was then placed into a plastic rat brain matrix and was coronal sectioned into 2 mm slices. The samples were stained with 2% 2,3,5-triphenyltetrazolium (TTC) solution in a 37°C for 20 min and were fixed with 10% formalin solution. The cerebral infarction areas of the first six sections from the frontal lobe were measured using an image analysis system (Image-Pro Plus 6.0 Media Cybernetics, USA). The ratio of infarct area to total brain area in each section of the rat brain was calculated, and the data were expressed as a percentage (%).

### 2.6. Assessment of neurological functions

The effects of GFW on neurological deficit score after ischemia for 2 h and following by reperfusion for 24 h in hyperglycemic rats with I/R brain injury were assessed as described according to a previous report [35]. The degree of neurological deficit is rated 0-5: 0 represents no neurological dysfunction and 1 represents contralateral forelimb consistently flexed. 2 represents decreasing grip ability on the contralateral forelimb with tail pulled and 3 represents spontaneous movement in all directions but circling toward contralateral side when pulled by the tail. 4 represents nonspontaneous walking with drowsiness, unconsciousness and 5 represents death.

### 2.7. TUNEL assay and immunohistochemical stain

Rats were deeply anesthetized by injection of zoletil^®^ (50 mg/ kg, i.p.) on day 22 after STZ injection. Intracardial perfusion with 200 *ml* of 0.9% saline followed by 4% paraformaldehyde in 0.1M PBS was performed before animals were decapitated. Brains were removed, immersed in 10% paraformaldehyde, sectioned at 2-mm intervals using a rodent brain matrix slicer (RBM- 4000C; ASI Instruments; Warren, MI), processed and embedded in paraffin, cut 2.5 μm thick on a microtome and processed for TUNEL and immunohistochemical staining. TUNEL assay of brain sections used Apo-BrdU-IHC^TM^ *In Situ* DNA Fragmentation Assay Kit (BioVision, Milpitas, CA). Incubated the brain slices with proteinase K for 20 min followed by 3 % H2O2 in methanol for 5 min to inactivation of endogenous peroxidase. TdT was added at room temperature and incubated overnight. Dark brown color indicated DNA breaks after incubation with DAB (3, 3’- diaminobenzidine tetrachloride) and hydrogen peroxide, followed by counterstaining with methyl green. Percentages of positive TUNEL staining cells within brain areas were estimated.

Brain slices were incubated with the anti-caspase-3 antibody (sc-7148, dilution 1:200, Biotechnology, Inc.; Santa Cruz, CA) overnight and immunohistochemical labeled by NovoLink Polymer Detection System Kit (Leica Microsystems Inc., Newcastle Upon Tyne, UK). Ratios of caspase-3-positive cells within brain areas were estimated.

### 2.8. Determination of expression of caspase-3, Bax and Bcl-2 by Western blot

Injection of zoletil^®^ (50 mg/kg, i.p.) sacrificed rats for biochemical studies. Brain tissues were quickly removed, and the cerebral cortex and hippocampus were separated on the ice. A 10% homogenate was prepared in lysis buffer, centrifuged at 12,000 (rpm) for 30 min at 4°C. Use the BCA protein assay kit to determine the samples protein concentration. Seventy mg protein was separated on 10% sodium dodecyl sulfate-polyacrylamide gels (SDS-PAGE) and transferred to polyvinylidene difluoride (PVDF) membranes. Membranes were incubated for one hr with 5% dry skim milk in TBST buffer at room temperature to block non-specific binding, then with the anti-Caspase-3, anti-Bax, anti- Bcl-2, anti-β-actin antibodies. Later, membranes were incubated with alkaline-phosphatase-conjugated secondary antibody for one hour at room temperature; bands visualized with chromogenic substrate 5-bromo-4-chloro-3-indolyl phosphate in the presence of nitroblue tetrazolium.

### 2.9. Cell culture

Murine BV-2 microglial cells were kindly provided by Professor Jau-Shyong Hong (Neurobiology Laboratory/Neuropharmacology group, NIEHS/NIH, NC, USA). BV-2 microglial cells were cultured in Dulbecco’s modified Eagle medium (DMEM) supplemented with 10% FBS, 100 units/*ml* penicillin, and 100 mg/*ml* streptomycin, and kept at 37°C in a humidified incubator with 5% CO2 and 95% air.

### 2.10. MTT assay

The BV-2 microglial cells were used to explore the effects of GFW on LPS-induced inflammation. The cells were cultured in a 96-well plate at the density of 5 × 10^4^ cells/well. BV-2 microglia cell cultures were administered with GFW and LPS for 24 h. MTT was added to each well, and the cells were incubated for 1 h. After culture media were discarded, DMSO was added to dissolve the formazan dye and the optical density was measured at 570 nm.

### 2.11. Nitrite quantification

NO generation was measured by the accumulation of nitrite in the culture medium. The colorimetric assay was used to determine nitrite with Griess reagent. BV-2 cells (5 × 10^4^ cells per well) in 96-well plates in 200 *ml* culture medium were treated with LPS (0.5 μg/*ml*) for 24 hours. 100 μ*l* of the isolated supernatant was added with an equal volume of Griess reagent in the 96-well plates for 10 min at room temperature and light avoidance. Standard solution of sodium nitrite prepared in the cell-culture medium was used to determine nitrite concentrations. The absorbance at 570 nm was read using an Elisa reader (Triad LT, DYNEX Technologies Inc, VA). Each experiment was duplicated three times.

### 2.12. Western Blot analysis of iNOS and COX-2

Cell lysates were prepared in lysis buffer, and the protein concentrations were determined by Bio-Rad protein assay kit (Richmond, CA, U.S.A.). Samples of protein (50 μg) were electrophoresed using 10% sodium dodecyl sulfate-polyacrylamide gel electrophoresis (SDS-PAGE) and then transferred to nitrocellulose membrane. The iNOS, COX-2 were assayed by specific antibodies (Santa Cruz Biotech, Santa Cruz, CA, U.S.A. Cell Signaling Technology). An enhanced chemiluminescence detection kit was used in immunodetection (Amersham, Piscataway, NJ).

### 2.13. Statistical analysis

Data were expressed as the mean ± standard error. For single variable comparisons, Student’s t-test was used. For multiple variable comparisons, data were analyzed by one-way ANOVA followed by Dunnett’s test. *P* < 0.05 was considered significant.

## 3. Results

### 3.1. Effects of GFW on cerebral infarct volume and neurological function in STZ-induced hyperglycemic rats with I/R brain injury

**Figure 2:**
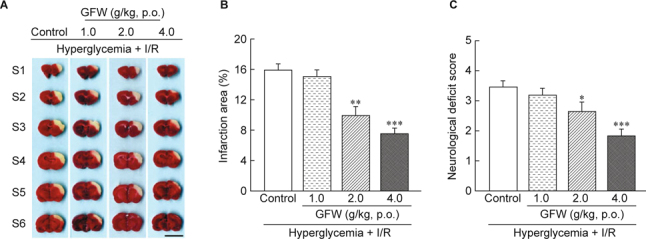

After blocking blood flow of both common carotid arteries and right cerebral artery for 90 min, all rats developed cerebral infarction after 24 h reperfusion: visibly white and non-infarction areas red-purple by TTC staining (Fig. 2A). GFW decreased infarct volume in a dose-dependent manner (Fig. 2B). The average percentage of infarct area in STZ-induced hyperglycemic rats was 15.96 ± 0.76 %. The average percentages of infarct area treated with different dosages of GFW (1, 2, 4 g/kg) in hyperglycemic rats with I/R brain injury were inhibited by 15.06 ± 0.88 %, 9.89 ± 1.21% (*P* < 0.01) and 7.59 ± 0.69 % (*P* < 0.001), respectively. The effects of GFW (1, 2, and 4 g/kg, p.o.) on neurological deficit score were assessed. Results showed that GFW significantly reduced neurological deficit score in hyperglycemic rats with I/ R injury (Fig. 2C). GFW (1, 2, and 4 g/kg, p.o.) improved neurological deficit in the STZ-induced hyperglycemic rats with I/R brain injury by 3.18 ± 0.23 (1. g/kg), 2.64 ± 0.31 (2 g/kg) and 1.82 ± 0.23 (4 g/kg), respectively.

### 3.2. Effects of GFW on brain apoptosis in hyperglycemic rats with I/R brain injury by TUNEL stain and on caspase-3 expression in immunohistochemical staining

**Figure 3:**
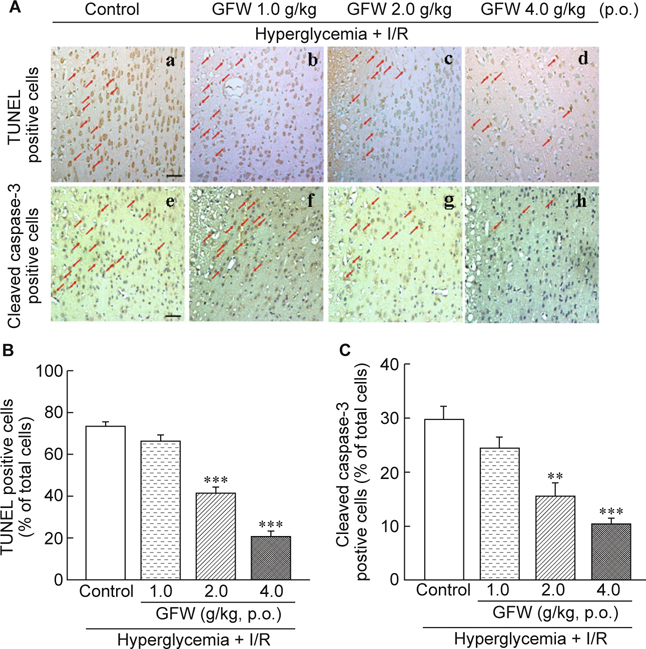

We used TUNEL to assay nucleosomal DNA fragmentation and on caspase-3 expression in immunohistochemical staining in the ischemic penumbra. Fig. 3 plots representative histological view of TUNEL stains and immunohistochemical staining in hyperglycemic rats. Treating with GFW (2 and 4 g/kg) observably decreased TUNEL-positive cells (Fig. 3B) and caspase-3 positive cells (Fig. 3C) in the hyperglycemic rats with I/R brain injury.

### 3.3. Effects of GFW on the expression of caspase-3, Bax and Bcl-2 in hyperglycemic rats with I/R brain injury

**Figure 4:**
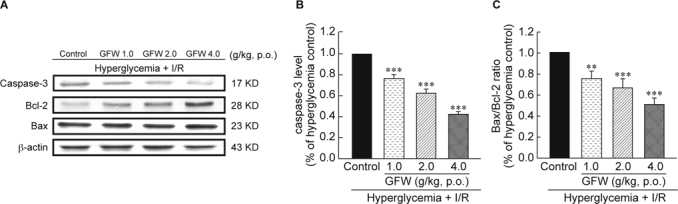

Fig. 4A showed the levels of protein expression as measured by Western blot. GFW could suppress caspase-3 expression in hyperglycemic rats with I/R brain injury in a dose-dependent manner (Fig. 4B, *P* < 0.001). GFW did not change Bax protein expression, but significantly enhanced Bcl-2 protein level. The Bax/Bcl-2 ratio was showed in Fig. 4C.

### 3.4. Effects of GFW on microglial cell viability, and NO production, iNOS, and COX-2 expression

**Figure 5:**
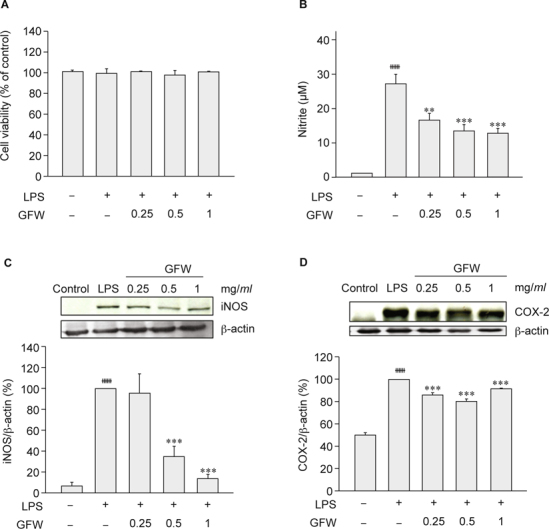

Fig. 5 showed the effect of GFW (0.25, 0.5, 1 mg/*ml*) on microglial cell viability, and NO production, iNOS and COX-2 expression by Western Blot analysis. GFW (0.5, 1 mg/*ml*) significantly reduced NO production (Fig. 5B, *P* < 0.01, *P* < 0.001, respectively) without affecting the BV-2 cell viability (Fig. 5A). How ever, GFW could attenuate the expression of iNOS (Fig. 5C) and COX-2 (Fig. 5D) in 0.5 μg/*ml* LPS-challenged BV-2 microglial cells.

## 4. Discussion and conclusion

Cerebral ischemia and infarction are commonly associated with atherosclerotic disease and stroke in carotid and vertebrobasilar circulations [1]. Ischemic lesions divide core zone (ischemic core) and penumbra (ischemic penumbra); the latter includes the area as a mild-to-moderate reduction of cerebral blood flow. The core area is very low cerebral blood flow and produces irreversible neuronal damage [36]. Necrosis occurs mainly in the core area, apoptosis mainly distributed in the penumbra [37-39]. Cases of ischemic stroke changed penumbra gradually. The presence of high blood sugar is a significant increase in the degree of damage to the ischemic brain penumbra [40, 41]. This study focused on penumbra and assessed GFW efficacy on apoptosis in STZinduced hyperglycemic rat brains after I/R injury, and determining apoptosis-related proteins in the penumbral area. In previous studies, stress-induced hyperglycemia is a modifiable risk factor for brain damage and associated with high mortality after stroke [6, 42]. Through diverse biochemical mechanisms, increased blood glucose concomitant with cerebral vascular injury may facilitate ischemia/reperfusion damage [43]. From the experimental results, infarcted brain tissue and noninfarcted brain tissue could be clearly observed on boundaries. Infarct area in STZ-induced hyperglycemia markedly increased.

As mentioned earlier, neurological deficit caused by I/R brain injury was associated with cerebral infarction size [44]. This study assessed neurological function: brain infarct was significantly reduced in hyperglycemic rats with I/R brain injury while treated with GFW. Greater vulnerability of the central nervous system in carotid artery ligation-induced cerebral ischemia means volume reduction of perceptual information, with disordered adaptation to environmental conditions and reproduction of conditioned reflexes [45].

Hyperglycemia-induced myocardial apoptosis also led to diabetic cardiomyopathy *via* mitochondrial cytochrome c-mediated caspase-3 activation pathway [25]. Many studies have suggested apoptosis playing an important role in cerebral ischemic pathogenesis. Diabetes following middle cerebral artery occlusion may increase the development of cerebral infarct injury and enhance apoptotic activity [46]. TUNEL stain is a standard method of DNA fragmentation associated marker with caspase-3 as another marker of apoptosis. In the Bcl-2 family, determination of antiapoptotic proteins like Bcl-2 and pro-apoptotic ones like Bax can expedite understanding of apoptotic mechanism. Caspase-3 activation and alteration in the expression of Bax/Bcl-2 were evident in the hyperglycemic rats [47-49]. Induction of diabetes causes hippocampal neuronal cell death in which apoptosis is associated with an increasing Bax/Bcl-2 ratios as well as caspase-3 level [50]. In our previous study, hyperglycemia-induced apoptosis correlated with an increase in Bax/Bcl-2 ratio, and increased caspase-3 activity [32]. In this study, TUNEL and immunohistochemical stains averred GFW was significantly reducing TUNEL positive cells and caspase-3 protein expression in hyperglycemic rats with cerebral ischemia/perfusion injury. Western blot also showed GFW substantially attenuating caspase-3 expression and elevating Bcl-2 expression, with Bax expression unchanged. A reduced Bax/Bcl-2 ratio in STZ-induced hyperglycemia with cerebral I/ R brain injury which portends GFW reducing cerebral ischemia, infarction, and neurological deficit, possibly *via* a decrease in apoptosis. This result concurs with prior studies [47].

Traditional herbal remedies in use for thousands of years help to prevent and treat several diseases: *e.g*., diabetes, stroke. For human health, they are still valuable and widely accepted due to various biological activity and low toxicity. GFW significantly attenuated infarct volume, neurological deficit and numbers of TUNEL as well as cleaved caspase-3 positive cells. This study is the first report to address the neuroprotective effects of GFW on apoptosis in the STZ-induced hyperglycemic rats with I/R injury.

Microglia and ischemic stroke is the double-edged sword [51]. An inflammatory response initiates within a few minutes to hours after ischemic stroke. Followed by microglia, astrocytes activation, and the production of chemoattractants, cytokines, chemokines [52-54], and subsequent infiltration of leukocytes [55, 56]. Microglial activation is the initial step of the inflammatory process in minutes [53, 57]. Two to three days following ischemia, the activation, and amplification of microglia reach the peak and continue for several weeks [58, 59]. Activated microglia, changes shape, and phenotype have the potential to phagocytose the presenting antigens, produce cytokines and matrix metalloproteinases that disrupt the blood-brain barrier [56]. Peripheral leukocytes infiltrate into the brain, further exacerbate inflammation and brain damage [51]. This study revealed GFW attenuated the infarct area of ischemic brain damage in STZ-induced hyperglycemic rats and inhibited microglial activation in LPS-induced BV-2 cells.

Based on our research data, we suggest that the neuroprotection of GFW in cerebral ischemia of STZ-induced hyperglycemic rats may be partly due to inhibition of Bax/Bcl2, caspase-3 signaling pathway, and neuroinflammation.
